# Timing of Antibiotic Prophylaxis in Elective Caesarean Delivery: A Multi-Center Randomized Controlled Trial and Meta-Analysis

**DOI:** 10.1371/journal.pone.0129434

**Published:** 2015-07-06

**Authors:** Chuan Zhang, Lingli Zhang, Xinghui Liu, Li Zhang, Zhiyou Zeng, Lin Li, Guanjian Liu, Hong Jiang

**Affiliations:** 1 Department of Pharmacy, Evidence-Based Pharmacy Center, West China Second University Hospital, Sichuan University, Chengdu, Sichuan, China; 2 Department of Obstetric & Gynecologic, West China Second University Hospital, Sichuan University, Chengdu, Sichuan, China; 3 Key Laboratory of Birth Defects and Related Diseases of Women and Children (Sichuan University), Ministry of Education, Chengdu, Sichuan, China; 4 Department of Pharmacy, Nanchong Central Hospital, Nanchong, Sichuan, China; 5 The Chinese Cochrane Center, West China Hospital, Chengdu, Sichuan, China; 6 Department of Obstetric & Gynecologic, Suining Central Hospital, Suining, Sichuan, China; Shanghai 1st Maternity and Infant Hospital of Tongji University, CHINA

## Abstract

**Objective:**

To compare the effectiveness of antibiotic prophylaxis before skin incision with that after umbilical cord clamping in elective caesarean delivery.

**Methods:**

We conducted a randomized open-label controlled trial with two parallel arms at three hospitals in western China. Participants meeting the inclusion criteria received antibiotics 30-60 minutes before skin incision while others received antibiotics after umbilical cords clamping. For the meta-analysis, studies were identified from the database of PUBMED, Cochrane Library and EMbase and assessed using the Cochrane risk of bias tool.

**Results:**

Four hundred and ten patients were randomized to receive antibiotics before skin incision (n = 205) or after umbilical cords clamping (n = 205). There was no difference in the incidence of postpartum endometritis (RR = 0.34, 95% CI 0.04 to 3.24), wound infection (RR = 3.06, 95% CI 0.13 to 74.69) and total puerperal morbidity (RR = 1.02, 95% CI 0.47 to 2.22). No increase in the incidence of neonatal sepsis (RR = 0.34, 95% CI 0.04 to 3.24), septic workup (RR = 0.41, 95% CI 0.08 to 2.07), or intermediate NICU admission (RR = 0.73, 95% CI 0.24 to 2.26) was observed. The meta-analysis involving nine RCTs showed that no statistically significant difference was found in terms of the risk of postpartum endometritis (RR = 0.73, 95% CI 0.39, 1.36), wound infection (RR = 0.80, 95%CI 0.55, 1.17), or puerperal morbidity (RR = 0.89, 95% CI 0.70, 1.13). No increase in the incidence of neonatal sepsis (RR = 0.65, 95% CI 0.35 to 1.20), septic workup (RR = 0.88, 95% CI 0.50 to 1.54), or intermediate NICU admission (RR = 0.91, 95% CI 0.70 to 1.18) was observed.

**Conclusion:**

For elective caesarean delivery, the effects of antibiotic prophylaxis before skin incision and after umbilical cord clamping were equal. Both antibiotic prophylaxis before skin incision and that after umbilical cord clamping were recommended for elective caesarean delivery. The outcome of further studies should address both maternal and neonatal infectious morbidity as well as long-term neonatal follow up.

**Trial Registration:**

Chinese Clinical Trial Registry ChiCTR-TRC-11001853

## Introduction

China had the highest overall incidence of caesarean delivery (CD) in Asia according to the WHO Global Survey On Maternal And Perinatal Health 2007–08 [1]. Ministry of Health of the People's Republic of China has appealed to reduce the incidence of CD and meanwhile they are exploring ways to decrease the risk of CD. Infections, such as endometritis and wound infections, were the most common complications in CD. Since there was overwhelming evidence for the effectiveness and necessity of prophylactic antibiotics during CD [2], the current debate focuses on the choice of antibiotics and the timing of administration [3].

American Congress of Obstetricians and Gynecologists (ACOG) [4] and The Society of Obstetricians and Gynecologists of Canada (SOGC) [5] recommended that antibiotic prophylaxis should be administered within 60 minutes before beginning CD, based on a meta-analysis which concluded that antibiotic prophylaxis before skin incision, comparing with after cord clamping, decreased the incidence of postpartum endometritis and total infectious morbidities, without affecting neonatal outcomes [6]. But Ministry of Health of China, in contrast to other countries, recommended antibiotic prophylaxis after cord clamping in CD instead of that before skin incision due to lack of evidence from trials in Chinese.

Three of meta-analysis strongly suggested that antibiotic prophylaxis given before skin incision for cesarean delivery, rather than after cord clamping, decreases the incidence of postpartum endometritis and total infectious morbidities, without affecting neonatal outcomes [6–8]. However, the current studies had two main limitations. First, they failed to distinguish elective CD from emergency CD, which leads to a clinical heterogeneity. Second, previous studies focused on the immediate impact on newborns but failed to address outcomes about growth and development of newborns, for example, colonization with commensal microorganisms in neonate [9–10].

Thus, we performed a multi-center randomized controlled trial to explore the following issues:

1. Is antibiotic prophylaxis before skin incision more effective than administration after cord clamping in reducing puerperal infectious morbidity in elective CD?
2. Does antibiotic prophylaxis administration before skin incision change neonatal gastrointestinal micro-ecological environment such as gut flora?

## Methods

### Ethics Statement

The trial was approved by Chinese Ethic Committee of registering clinical trials and registered in Chinese Clinical Trial Registry with a registration number of ChiCTR-TRC-11001853 (S1 File). Patients gave their written informed consents.

### Design

This multi-center randomized controlled trial with two parallel arms was conducted at three hospitals in western China (West China Second University Hospital, Nanchong central hospital and Suining central hospital), from January 1st, 2012, through to June 30th, 2013. Random sequence was generated with SPSS 16.0-generated algorithm by an investigator (LGJ) without involvement in the trial conducting. Participants were assigned randomly to receiving antibiotics before skin incision or after cord clamping with a 1:1 ratio. Treatment assignments, kept in sealed opaque envelopes with only labeled numbers, were revealed after the investigator (ZL) evaluated participants and confirmed the eligibility. Another investigator (LL) recorded patient enrollment and patient assignment in each group. The research assistant (CMH) and the statistician (LGJ), who were responsible for outcome recording and data analysis, respectively, were blinded to treatment assignment. The Trial protocol was in S2 File. The case report form was in S3 File.

### Participants

Subjects were eligible and were included in the elective CD group before labor if their gestational weeks were more than 37 weeks. Exclusion criteria included cephalosporin allergy, exposure to any antibiotic agent two weeks before CD, the temperature above 37.5°C before CD, concomitant premature rupture of membrane, pernicious placenta praevia or the need for emergent cesarean delivery.

### Interventions

Two grams of cefathiamidine (Guangzhou baiyunshan pharmaceutical Co. LTD), of which the predominant effect was against gram positive organisms and the pharmacokinetic properties were similar to those of cefazolin, dissolved in 100 ml saline solution were used for antibiotic prophylaxis during 0.5–2 hours before skin incision or after umbilical cord clamping according to the determined allocation. Another two grams of cefathiamidin were given 6 hours after CD. Participants were followed till 6 weeks postpartum by telephone.

## Outcome

### Primary Outcome

Endometritis was diagnosed when maternal temperature was above 38°C in two separate occasions accompanied with uterine tenderness, tachycardia, or leukocytosis. Surgical site infection was diagnosed when there was purulent discharge, erythema, and induration of the incision site. Puerperal morbidity was defined as any condition when maternal temperature was above 38°C on two separate occasions due to infection.

Neonatal sepsis was diagnosed by a positive blood culture. Organism, antibiotic resistances, and clinical course data were recorded. Sepsis workup and the place of admission of all neonates were determined by the neonatologists who were blinded to group assignment.

### Secondary Outcome

The first stool of newborns after birth was obtained and was examined by clinical laboratory. The results were recorded by the trial assistant (CMH). The gut flora of the neonates was examined with the method of Direct Rapid Smear and the results were classified as normal, mild abnormal, moderate abnormal and serious abnormal. Normal was defined as a situation in which Gram-positive and negative bacilli and cocci were within the normal range. Mild abnormal was defined as the reduction of Gram-positive bacteria and the proliferation of Gram-negative bacilli or positive cocci. Moderate abnormal was defined as the significant reduction of Gram-positive bacteria, proliferation of Gram-negative bacilli or positive cocci, and inverse ratio of bacilli and cocci. Serious abnormal was defined as a situation in which only one dominant bacteria or fungi was observed and other floras were suppressed [11].

### Statistical Analysis

Power was calculated by using incidence of maternal infections which was reported to be 17% in the literatures [12]. Using a power of 0.80 and a two-tail alpha error of 0.05, we calculated and found that 190 subjects per arm were necessary for detecting a 50% decrease in overall infectious morbidity. Considering 10% loss of follow-up, a total of 410 patients were required, with 205 in each group.

Intention to treat (ITT) was used to analyze the data of trial. Statistical software SPSS 16.0 was used for analyses. Continuous parameter was analyzed with student’s t-test. Discrete data was analyzed with chi-square test. Ordered variables such as four categories of neonatal gut flora were analyzed with Wilcoxon rank sum test. Results with a p value<0.05 were considered statistically significant.

### Systematic review and meta-analysis

We searched PubMed, EMbase and Cochrane Library for studies published before June 2014 by using the following key words: (cesarean OR “CD”) AND (“timing of antibiotic” OR “prophylactic antibiotic” OR antibiotic). Randomized controlled trials about timing of prophylactic antibiotics in elective CD were included in the meta-analysis. Prophylactic antibiotics were administered before skin incision in the experimental group, while they were administered at the time of clamping umbilical cord in the control group. There was no limitation of the kind and dose of antibiotics. We only included studies published in English.

Data extraction form designed according to Cochrane Systematic Review Handbook (version 5.1.0) was used to extract the relevant information from each RCT independently by CZ and LZ. Bias assessment tool developed by Cochrane Collaboration was used to assess the risk of bias. We had considered that there might be risk of bias in the process of random sequence generation, allocation concealment, blinding of participants, blinding of assessment data collecting and result reporting.

Meta-analysis was conducted with Revman (version 5.1). Heterogeneity was estimated by χ^2^ test. The treatment effect was expressed as relative risks (RRs) with 95% confidence interval. If the heterogeneity is not significant (I^2^≤50%, P>0.1), the fixed effect model was used; otherwise, the random effect model would be used. We planned to carry out sensitivity analysis for the primary outcomes by restricting our analysis to trials assessed as having moderate risk of bias.

### Results of RCT

**Fig 1:**
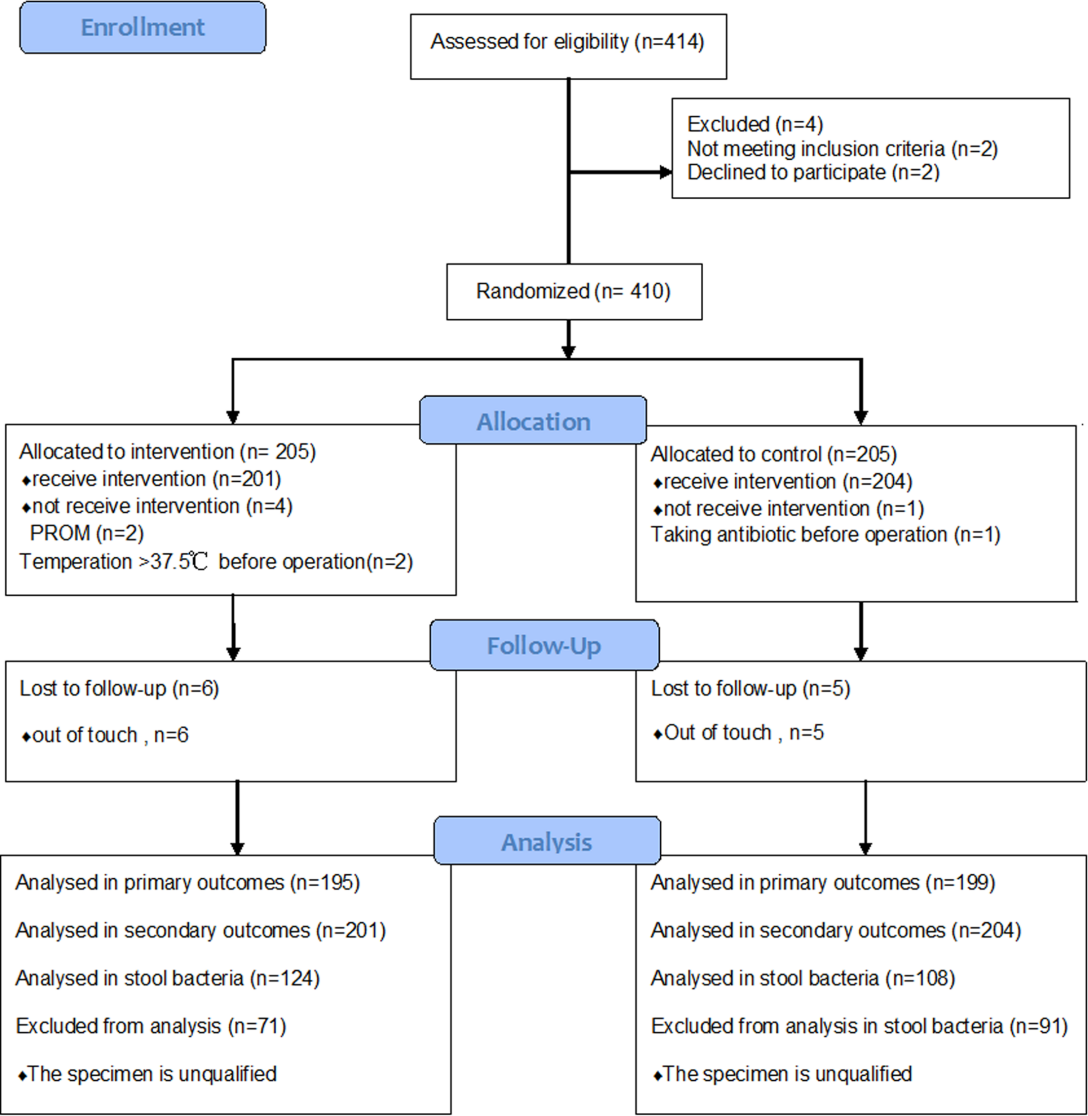
Trial flow diagram.

Totally 410 patients were eligible in this study. They were randomly allocated to the experimental group (received antibiotics before skin incision) and the control group (received antibiotics after cord clamping), with 205 in each group. In the experimental group, two patients got rupture of membranes just before CD and another two got upper respiratory infection before CD, as a result of that, 201 patients received antibiotics before skin incision. In the control group, because one patient got a fever before CD, 204 patients received antibiotics after cord clamping. In the phase of follow-up, 6 patients from the experimental group could not be contacted, and thus 195 received a telephone interview. Comparatively, 5 patients from the control group could not be contacted, so 199 received a telephone interview. Trial flow chart is presented in (Fig 1)

Characteristics of patients are presented in Table 1. There were no significant differences between the two groups except operation time, which was shorter in experimental group. The mean time between antibiotic administration and incision was 35.6 ±15.2 mins.

**Table 1:** Characteristics of patients.

Characteristic	Experimental group (n = 205)	Control group (n = 205)	P-value
Age, (years)	30.33±4.54	29.9±4.51	0.38
Temperature, (°C)	36.56±0.27	36.54±0.27	0.41
Body mass index (kg/m^2^)	27.25±3.2	27.29±2.9	0.91
Pulse(sequence/min)	86.66±10.9	85.93±10.15	0.49
Breath(sequence/min)	28.2±7.76	29.2±8.87	0.44
Gestation (weeks)	38.82±0.90	39.05±0.92	0.012
Length of stay(days)	5.74±1.99	5.59±1.75	0.43
Operation time (minutes)	38.35±11.3	41.63±11.11	0.004
Blood loss (ml)	263±100.72	249±100.2	0.17
Epidural anesthesia (%)	198 (96.58%)	199 (97.07%)	1.0
Indication for CD: repeated scars	31	40	0.92
Indication for CD: Low-lying placenta	85	78	0.92
Indication for CD: Patients’ choice	89	87	0.92

### Primary Outcomes

195 patients in the experimental group and 199 patients in the control group were analyzed with respect to maternal primary outcomes. The results were presented in Table 2.

**Table 2:** Primary outcomes.

		Experimental group (n = 195)	Control group (n = 199)	P- value
**Maternal**				
	Endometritis	1 (0.5%)	3 (1.5%)	0.62
	Surgical site infection	1 (0.5%)	0	0.5
	Urinary tract infection	0	0	-
	Puerperal morbidity	12 (6.15%)	12 (6.03%)	0.98
**Neonate**				
	Sepsis	1(0.5%)	3 (1.65%)	0.37
	Septic workup	2(1.08%)	5 (2.7%)	0.28
	NICU admission	5 (2.7%)	7(3.8%)	0.57

**NICU:** Neonatal intensive care unit

The incidence of endometritis was 0.5% in the experimental group and 1.5% in the control group; however, this difference did not research statistical significance (RR = 0.34, 95% CI 0.04 to 3.24). There was no statistical difference in the incidence of wound infection (RR = 3.06, 95% CI 0.13 to 74.69) and total puerperal morbidity (RR = 1.02, 95% CI 0.47 to 2.22).

Variables are illustrated in Table 2. There were no significant differences observed between the two groups in neonatal sepsis (RR = 0.81, 95% CI 0.48 to 1.36), sepsis workups (RR = 0.41, 95% CI 0.08 to 2.07) and NICU admission (RR = 0.72, 95% CI 0.23 to 2.25) in the randomized controlled trial.

### Secondary Outcome

There was no difference in neonatal stool bacterial flora Table 3.

**Table 3:** Secondary outcomes.

	Experimental group (n = 124)	Control group (n = 108)	P- value
Normal	14	15	0.48
Mild abnormal	58	40	
Moderate abnormal	25	28	
Serious abnormal	27	25	

### Meta-analysis of RCTs

**Fig 2:**
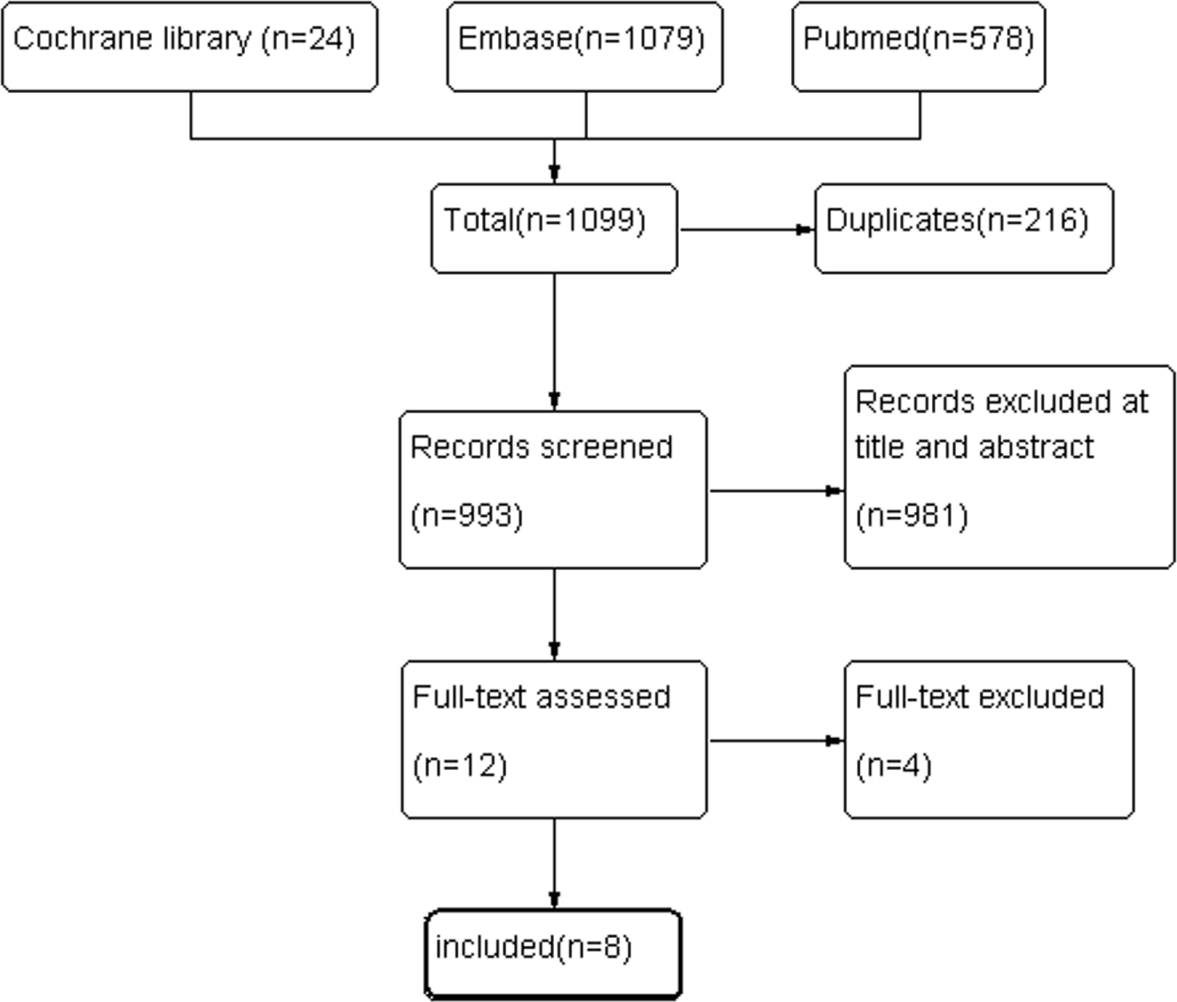
Flow diagram of included and analysed randomized controlled trials.

Flow of study selection was shown in (Fig 2). After removing the duplicate records, a total of 993 studies were screened based on titles and abstracts. Totally 962 studies were excluded because they were not RCTs or they failed to enroll patients with CD. The full text of the remaining 12 studies was checked, and a further four were eliminated because patients with emergency CD were enrolled. We also included our RCT. Finally, nine RCTs were included in the meta-analysis.

Details of risk of bias assessment were shown in Table 4. Eight RCTs adequately described generation of random sequence and allocation. Three studies had both doctors and patients blinded, two studies had doctors or outcome assessment blinded, and four RCTs did not have anyone blinded. Five studies reported the number of patients who caused loss to follow up. All studies appeared to have no selective reporting. Finally, five studies were judged as having moderate risk of bias and four RCTs were judged as having high risk of bias.

**Table 4:** Risk of bias of included studies in the meta-analysis.

Study (year)	Random sequence generation	Allocation	Blinding of participants	Blinding of assessment	Incomplete outcome	Selective reporting	Risk of bias
Nokiani (2009) [13]	Unclear	Unclear	Doctors Patients	Unclear	Unclear	No	High
Yildirim (2009) [14]	Unclear	Block random using sealed, sequentially distributed envelopes to which the letters A and B had been allocated	Not blinding	Unclear	Unclear.	No	High
Macones(2011) [9]	Permuted blocks	Unclear	Doctors	Unclear	Unclear	No	High
Witt (2011) [10]	A study nurse checked the randomization list and handed the appropriate infusion bag to the anesthesiologist.	Unclear	Doctors and patients	Unclear	32 (4.3%) women were lost to follow-up	No	Mederate
Osman (2012) [15]	Computer generated block-randomization	Concealed envelope system was used to allocate the patients	Not blinding	Yes	Unclear	No	Mederate
Francis (2013) [16]	The randomization sequence was generated by the hospital biostatistician.	The randomization list was e-mailed to the research pharmacist, who was the only person with access to the randomization information	Doctors and patients	Unclear	95 women (10.6%) were lost to follow-up	No	Mederate
Kalaranjini(2013)[17]	Unclear	The patients were randomly categorized into two groups using serially numbered opaque sealed envelope technique	Not blinding	Unclear	Unclear	No	High
Kandil (2014) [18]	Unclear	Fifty cards were prepared for each intervention. All the cards were inserted into opaque envelopes then were shuffled to produce a form of random assignment. The envelopes were sequentially numbered according to their final arrangement.	Not blinding	Unclear	Unclear	No	High
Zhang 2013	Computer-generated randomization sequence assigned participants into two treatment groups	Allocation was concealed in sealed, sequentially numbered, brown envelopes (opaque)	Not blinding	Yes	16 women (3.9%) were lost to follow-up	No	Mederate

**Fig 3:**
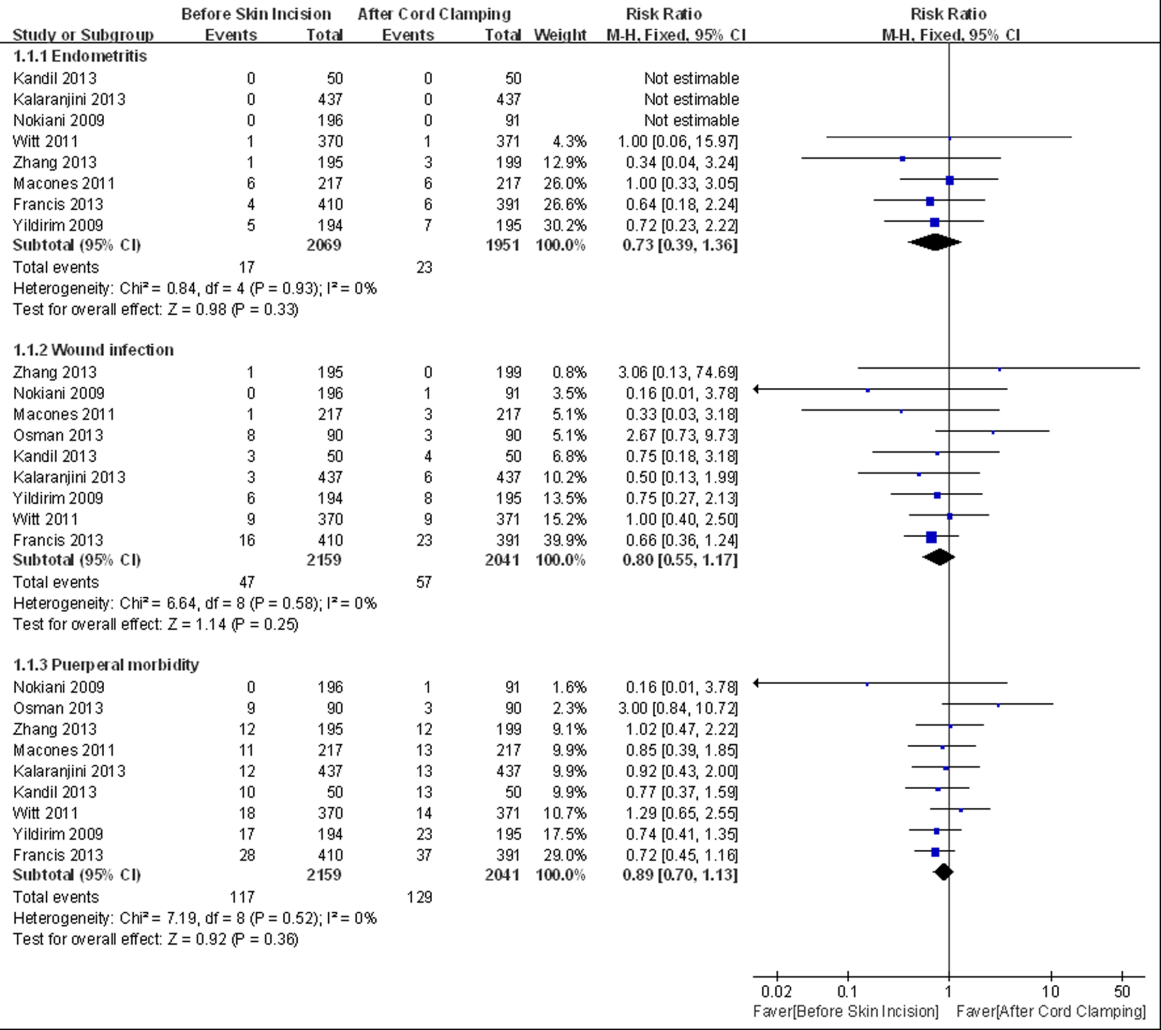
Meta-analysis of maternal primary outcomes.

**Fig 4:**
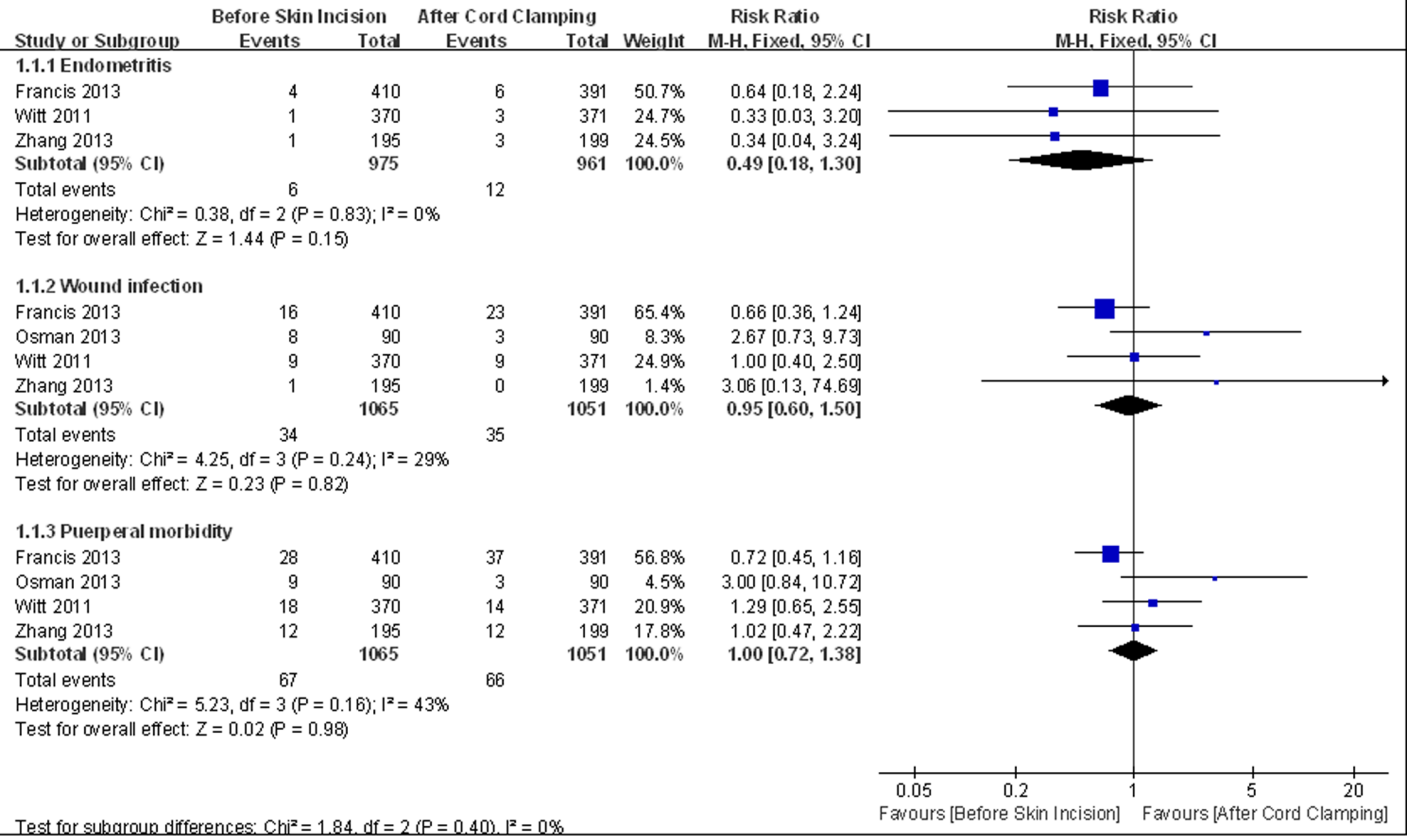
Sensitivity analysis of maternal primary outcomes.

Meta-analysis was conducted based on nine trials, including 2159 patients in the experimental group and 2041 patients in the control group. There was no difference in the incidence of endometritis (RR = 0.73, 95% CI 0.39 to 1.36, HeterogeneityI^2^ = 0%), wound infection (RR = 0.80, 95% CI 0.55 to 1.17, HeterogeneityI^2^ = 0%) and total infectious morbidity (heterogeneityI^2^ = 0%, RR = 0.89, 95% CI 0.70 to 1.13, HeterogeneityI^2^ = 0%) (Fig 3). The sensitivity analysis did not show significant change in the pooled effects (Fig 4).

**Fig 5:**
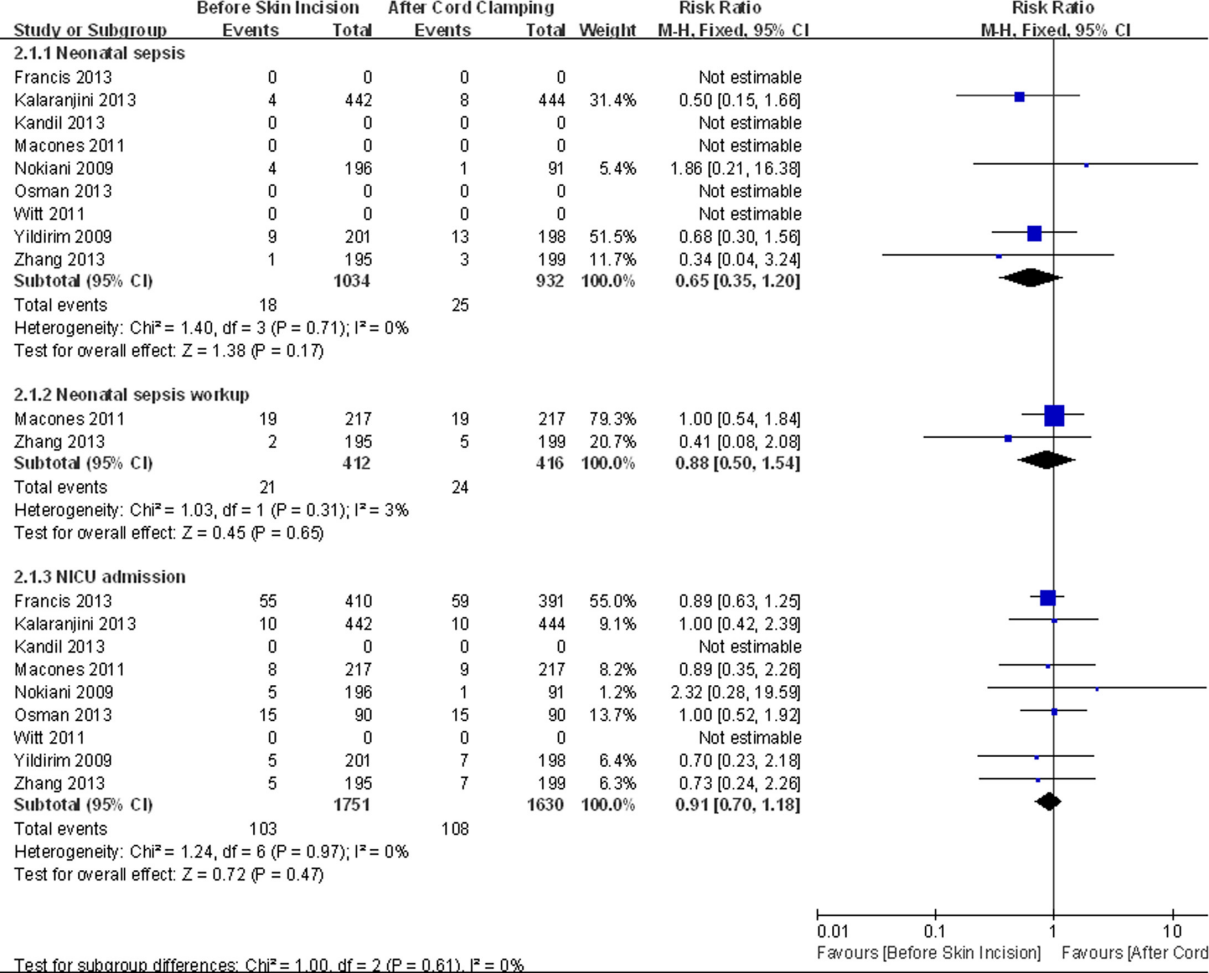
Meta-analysis of neonatal primary outcomes.

**Fig 6:**
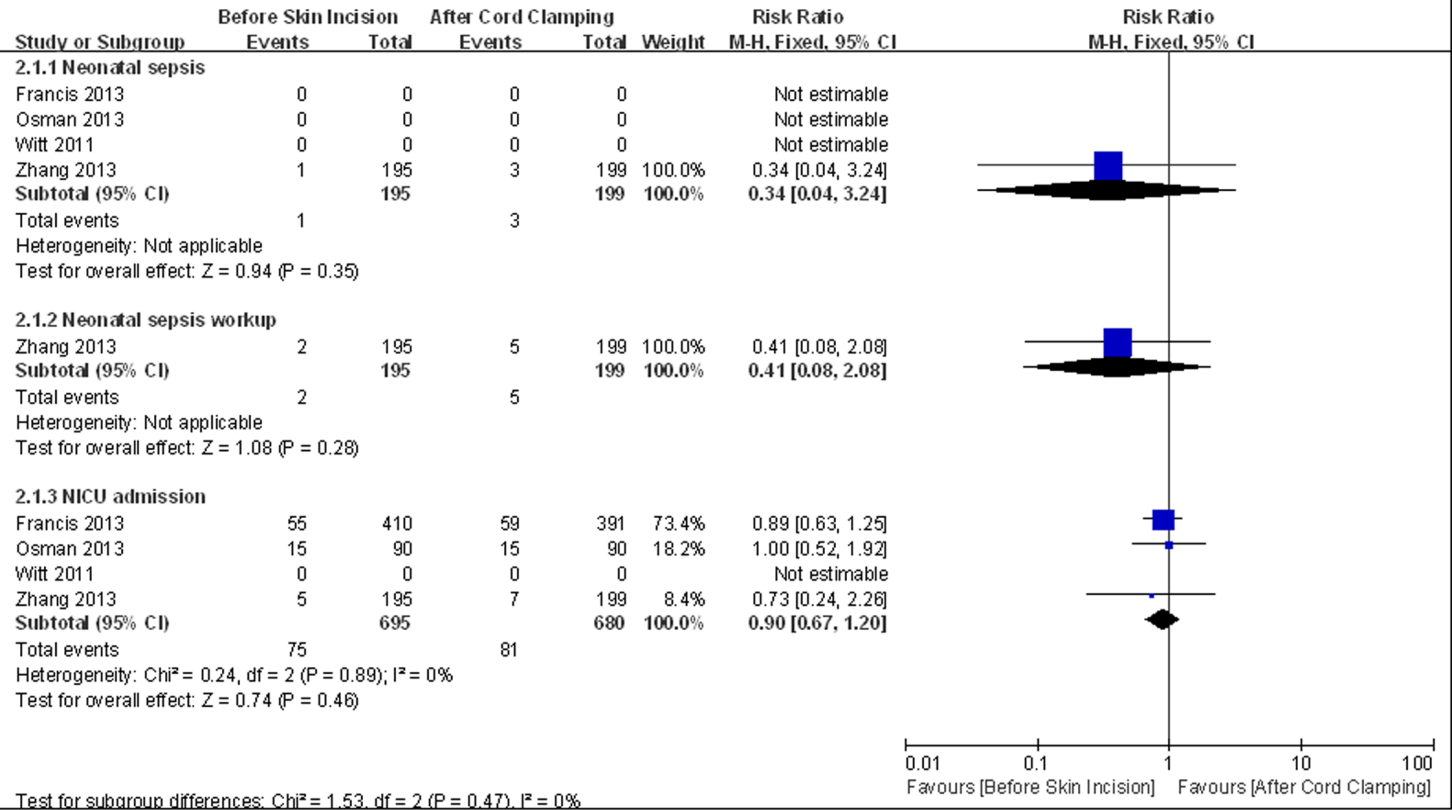
Sensitivity analysis of neonatal primary outcomes.

Eight trials reported neonatal outcomes. Meta-analysis showed no significant difference between the two groups in neonatal sepsis (RR = 0.65, 95% CI 0.35 to 1.2, HeterogeneityI^2^ = 0%), sepsis workups (RR = 0.88, 95% CI 0.5 to 1.54, HeterogeneityI^2^ = 0%) and NICU admission (RR = 0.91, 95% CI 0.70 to 1.18, HeterogeneityI^2^ = 0%) (Fig 5). The sensitivity analysis did not show significant change in the pooled effects (Fig 6).

## Discussion

The results of this multi-center randomized controlled trial and meta-analysis suggest that antibiotic prophylaxis before skin incision, compared with that after cord clamping, did not affect the incidence of maternal infectious morbidity in elective CD. Likewise, administration timing of antibiotics did not have impact on neonatal outcomes, including neonatal sepsis, sepsis workup and NICU admission.

In 1961, Burke [19] demonstrated an animal model which showed that antibiotics given before contamination of the wound decreased the rate of infection. This led to the use of preoperative prophylactic antibiotics in almost all surgeries that require prophylaxis. However, in the case of cesarean delivery, the current debate focuses on the concern that preoperative antibiotic dosing is related to a substantial plasma level in the neonate [20]. This therapeutic drug level in the newborn may alter blood culture results, and thus, delay or mislead the diagnosis of neonatal sepsis and increase sepsis workup [20]. It was a common practice to delay antibiotics at the time of umbilical cord clamping until 2008 when a meta-analysis strongly suggested that antibiotic prophylaxis given before skin incision for cesarean delivery, rather than after cord clamping, decreases the incidence of postpartum endometritis and total infectious morbidities, without affecting neonatal outcomes [6]. Since then, two other meta-analyses have assessed maternal and neonatal infectious morbidity in women undergoing cesarean delivery receiving preoperative prophylaxis compared with those receiving after umbilical cord clamping [7–8]. Both of the two meta-analyses are in agreement with the previous reviews.

It's worth noting that three of the meta-analysis did not distinguish elective CD from emergency CD [6–8]. Postpartum morbidity was higher in emergency CD than in elective CD and vaginal delivery. Marcollet found that the relative risk of any postpartum complication (serious or minor) in women who were infected with HIV was increased by 1.85 (95%CI, 1.00–3.39) after elective CD and 4.17 (95%CI, 2.32–7.49) after emergency CD, compared with that after vaginal deliveries (P = .0001) [21]. Participants in our meta-analyses were limited to patients with elective CD. Therefore, this is one of the most important reasons why our results are inconsistent with those of previous studies.

However, neonates being influenced by early exposure to antibiotics not only masked sepsis in a macroscopic view but also hindered colonization with commensal microorganisms in intestines of neonates at the micro level. Colonization with commensal microorganisms occurs swiftly after parturition [22–23] in neonates. Gut microbiotas in infants will be influenced by the type of infant feeding and other factors including mode of delivery, gestation age, infant hospitalization and antibiotic treatment [24]. Cesarean delivery not only prevents the newborns from being exposed to bacteria in the birth canal but also decreases the general bacterial exposure from in the mother because of routine antibiotic prophylaxis in CD [25]. We proposed a hypothesis that exposure to antibiotics will eradicate colonization with commensal microorganisms in intestines of neonates. There was no significant difference between the two groups in neonatal faeces bacterial flora; however, the number of patients with moderate and serious abnormal faeces bacterial flora in the experimental group was more than that in the control group. Most recently, Elahi et al found the reason why newborn infants are highly susceptible to infection was that neonatal CD71^+^ cell-mediated protection against aberrant immune cell activation in the intestine was beneficial to colonization with commensal microorganisms. Conversely, circumventing such colonization by using antimicrobials will override these protective benefits [26]. Therefore, we inferred that early exposure to antibiotics may cause microscopic changes of neonates, for example, imbalance of colonized bacteria, influencing their long-term growth and development. We did not find significant difference in neonatal gut flora between the two groups. However, neonatal long-term growth and development should be paid attention in future studies.

Another debate focusing on caesarean delivery was the choice of antibiotic [3]. Hopkins conducted a meta-analysis concluding that both ampicillin and first-generation cephalosporins had similar efficacy in reducing the incidence of postoperative endometritis. There did not appear to be added benefit in utilizing a broader spectrum agent [27]. However, three studies conducted after the meta-analysis found that broad-spectrum antibiotics were associated with a statistically signiﬁcant reduction in infection rates, endometritis, and wound infection compared with narrow-range agents [28–30]. Conflicting results may be due to different patients who received elective or emergency CD. In our opinions, it is necessary to distinguish elective CD from emergency CD not only for timing of antibiotic prophylaxis, but also for choosing of antibiotics.

There are several limitations in our study that should be acknowledged. First, the sample size of the RCT was not large enough. We used a maternal infectious morbidity of 17% which is very high especially in the elective CD. The data from meta-analysis showed that the maternal infectious morbidity was 5% to 6%. However, the meta-analysis made up for the limitation because the number of patients in the meta-analysis reached 4200, of which the power is 99% to detect a 50% decrease in overall infectious morbidity of participants. Second, the antibiotic adopted in our RCT was cefathiamidine rather than cefazolin used in other studies. Different antibiotics may vary in effectiveness. Finally, the loss of follow-up rate in neonatal faeces bacterial flora was up to 43%, which may increase bias.

## Conclusion

Taken together, our results demonstrated that antibiotic prophylaxis before skin incision and after umbilical cord clamping were equivalent for elective caesarean delivery. Both antibiotic prophylaxis before skin incision and that after umbilical cord clamping were recommended for elective caesarean delivery. Further studies have to address both maternal and neonatal infectious morbidity as well as long-term neonatal follow up.

## Supporting Information

S1 File Ethical review report. (DOC)

S2 File Trial protocol. (DOC)

S3 File Case Report Form. (DOCX)

S4 File CONSORT 2010 Checklist. (DOC)

S5 File PRISMA Checklist. (DOC)
