# Supplementary material for: Timing of Antibiotic Prophylaxis in Elective Caesarean Delivery: A Multi-Center Randomized Controlled Trial and Meta-Analysis
Source: PLoS One. 2015 Jul 6;10(7):e0129434. doi: 10.1371/journal.pone.0129434 (PMC4492889; doi:10.1371/journal.pone.0129434)
Supplement: S1 File — (DOC) [file pone.0129434.s001.doc]

**Chinese Ethics Committee of Registering Clinical Trials**

**Ethical Review Report**

**Title**: Timing of perioperative antibiotics for caesarean: a multicenter randomized controlled study

**Applicant**: Lingli Zhang

**Telephone**: 028-85503054

**Applicant's institution:** West China Second University Hospital, Sichuan University

**Approved No. of ethic committee:** ChiECRCT-2011045

In accordance with *Ethical Review Methods for Biomedical Researches Involving Human Subjects (Trial)* (WKJF [2007] No. 17), the Committee implemented ethical review on *Multicenter Randomized Controlled Study on timing of antibiotic prophylaxis for caesarean* submitted by Zhang Lingli, Professor of West China Second University Hospital, Sichuan University.

After independent review, the experts from the Committee proposed some suggestions for revision. With the second review after revision, they considered that the revised research proposal and informed consent comply with the ethical standard for biomedical research; therefore, they agreed to implement the trial.

The research team was required to report to the Committee in case of any adverse event related to the research during and after the trial. The Committee would decide whether to suspend the trial according to the situation.

**Statement**

As a commonweal independent organization, the Chinese Ethics Committee of Registering Clinical Trials is only responsible for reviewing the ethics principle of clinical trial and scientificity of research design, proposing suggestions on feasibility of the trial and requiring the clinical trial to be registered at the Registration Center.

**Review contents and opinions**

1. Whether the qualification and experience of the researcher complies with trial requirements;
2. Whether the research approach complies with the requirements of ethics principle and scientificity;
3. Compared with the anticipated benefits of the research, whether the potential risks suffered by subjects are suitable;
4. Whether the related information, which is provided to the subjects (or their family members, guardians and legal representatives) during the procedures of informed consent, is complete and easy to understand and whether the way to obtain informed consent is proper;
5. Whether proper measures are taken to keep the confidentiality of subjects’ information;
6. Whether the inclusion and exclusion criteria for the subjects are proper and fair;
7. Whether the subjects are clearly informed of their rights, including the right of quitting the trial at anytime during the research, without giving any reason and being discriminated;
8. Whether the subjects receive reasonable compensation due to the research; for example, whether the treatment is correct and the compensation measures are proper when the subjects are injured, even dead, because of participating in the research;
9. Whether persons from researchers are specially designated to deal with issues concerning informed consent and safety of the subjects;
10. Whether proper protection measures are taken to prevent potential risks for the subjects during the research;
11. Whether there is any conflict of interest between researchers and subjects.

**Comprehensive opinions**

1. Research proposal: A randomized controlled trial is employed for the research. However, the proposal presents little information about the elements crucial for quality of the trial, such as the generation of random sequence, blind method and allocation concealment. Please supplement more information to make improvement.

2. Informed consent: A lot of necessary information is missed in the informed consent. There is no clear statement for some items, such as “Whether the subjects receive reasonable compensation due to the research; for example, whether the treatment is correct and the compensation measures are proper when the subjects are injured, even dead, because of participating in the research” and “Whether proper protection measures are taken to prevent potential risks for the subjects during the research”. In addition, the statement of “Participating in the research will not cause any risk to you” in the informed consent is obviously contrary to medical knowledge and does not conform to the ethics principle.

3. In the research proposal, the statistical method describes “the relative risk calculated and adjusted with logistic regression”. However, with logistic regression can only obtain odds ration, without relative risk. Please propose explanation or rectification. Besides, the statistical method is too simple to completely satisfy the current research design. Multilevel model and other methods are suggested to be employed to study real effect of repeated measurement data.

4. In the research proposal, the records on outcome measurement of the postoperative shall include time point of follow-up. Besides, follow-up does not only refer to tracking of the subjects after them leaving the hospital.

5. The format and contents of the informed consent do not conform to requirements. Please use the template provided by the Committee to make revisions.

6. Please provide research flow chart, including allocation method and time point of outcome measurement.

7. Please specify the allocation method.

8. Definition of adverse reaction and treatment measures for it is lacked. Some person must be specially designated to take charge of signing of informed consent and monitoring and treatment of adverse reaction.

9. At the end of the second paragraph of informed consent, after the sentence of “we will call you for your and your baby’s situation”, please add “if you agree to participate in the research, we hope you will try the best to cooperate with our follow-up”.

10. Should age range and pregnancy times in the past be taken into consideration for the inclusion and exclusion criteria?

11. Please supplement design details of random and blind methods.

12. Please supplement setting of the two phases (including time points of follow-up and measurement indicators).

13. Should length of stay be taken into the consideration? For example, if outcome measurement is the follow-up measurement at multiple time points, survival analysis can be adopted.

**Statement**

**The applicant has already revised the research proposal and informed consent according to the above problems and suggestions.**

**Members of the Committee: Dayong Wu, Jin Wen, Qin Wang, Xunzhe Yang, Minawar.** **Abdul, Xiaoyan Yang, Guanjian Liu**

**Experts for second review: Wu Taixiang, Minawar.** **Abdul**

**Secretary general of the Committee: Taixiang Wu**

**Chinese Ethics Committee of Registering Clinical Trials**

**January 5, 2012**
