# Supplementary material for: Timing of Antibiotic Prophylaxis in Elective Caesarean Delivery: A Multi-Center Randomized Controlled Trial and Meta-Analysis
Source: PLoS One. 2015 Jul 6;10(7):e0129434. doi: 10.1371/journal.pone.0129434 (PMC4492889; doi:10.1371/journal.pone.0129434)
Supplement: S3 File — (DOCX) [file pone.0129434.s003.docx]

**剖宫产抗生素预防使用时机的多中心随机对照试验**

**病例报告表**

**（Case Report Form）**

受试者姓名拼音缩写： □□□□

试验开始日期（time started）： □□□□年□□月□□日

试验结束日期（time completed）：□□□□年□□月□□日

**填表说明**

**(Introductions)**

1. 请用钢笔或签字笔填写，字迹应清晰，易于辨认。
2. 受试者姓名拼音缩写四格应填满，两字姓名按每个字前两个字母填写；三字姓名按每个字的首个字母和第三个字的第二个字母填写；四字姓名按每个字的首个字母填写。举例：张红 ZHHO，刘书名 LSMI，欧阳小惠 OYXH。
3. 在所有选择项目中，请在相应的方框中划“×”，如×。
4. 填写数字时应将□都填满，位数不够的靠右侧填写，左侧空出填“0”。如。
5. 在需填写文字的方框内，优先保证填写左边第一个方框，然后右边第一个方框；多余的方框内，请划“—”，如—。

6. 每项填写内容务必准确，不得随便涂改，如发现填写内容有误，应在原记录上划“﹨”，在旁边写明正确内容，并注名修改者及日期。不要用任何方式(橡皮、涂改液等)涂抹原记录。

7. 不要改变病例报告表的格式，如发现表中没有位置填写记录者希望记录的资料时，请将有关信息记录于后面的空白附页中。

8. 知情同意书一般为受试者签名。如受试者有特殊情况，可由受试者法定代理人签名。

9. 如果遇到紧急情况(如发生不良反应)时请及时其联系主管医生或试验人。

**试验观察流程图**

**(Trial flow chart)**

| 项目 | 入组前（天） | | 时间 | | | | | | | 停药后 | |
| --- | --- | --- | --- | --- | --- | --- | --- | --- | --- | --- | --- |
|  | -6～-1 | 0 | 手术当天 | 术后1天 | 术后2天 | 术后3天 | 术后4天 | 术后5天 | 术后6天 | 2周 | 6周 |
| 知情同意书 |  | **×** |  |  |  |  |  |  |  |  |  |
| 入组筛选 |  | **×** |  |  |  |  |  |  |  |  |  |
| 病例简况 |  | **×** |  |  |  |  |  |  |  |  |  |
| 一般情况检查 |  | **×** |  |  |  |  |  |  |  |  |  |
| 术中情况 |  |  | **×** |  |  |  |  |  |  |  |  |
| 用药记录 | **×** |  | **×** | **×** |  |  |  |  |  |  |  |
| 体温测量 | **×** | **×** | **×** | **×** | **×** | **×** | **×** | **×** |  |  |  |
| 血常规记录 |  |  |  |  |  | **×** |  |  |  |  |  |
| 小便分析记录 |  |  |  |  |  | **×** |  |  |  |  |  |
| 子宫内膜炎诊断记录 |  |  |  | **×** | **×** | **×** | **×** | **×** |  |  |  |
| 伤口查看记录 |  |  |  | **×** | **×** | **×** | **×** | **×** |  |  |  |
| 新生儿大便菌群比例 |  |  |  |  | **×** |  |  |  |  |  |  |
| 观察不良反应 |  |  | **×** | **×** | **×** | **×** | **×** | **×** | **×** | **×** | **×** |
| 电话随访 |  |  |  |  |  |  |  |  |  | **×** | **×** |

| **入组筛选** | | |
| --- | --- | --- |
| **纳入标准**  **Inclusion criteria** | | |
|  | 是 | 否 |
| 择期剖宫产 | □ | □ |
| 年龄18-40岁 | □ | □ |
| 孕周>37周 | □ | □ |
| 同意参加试验并签署知情同意书 | □ | □ |
| 以上任一答案为否，此受试者不能参加试验  **排除标准**  (Exclusion criteria) | | |
|  | 是 | 否 |
| 头孢硫脒过敏 | □ | □ |
| 术前两周使用抗生素 | □ | □ |
| 术前体温>37.5℃ | □ | □ |
| 妊娠合并前置胎盘，胎盘早剥，胎膜早破任一 | □ | □ |
| 参加其他临床试验 | □ | □ |
| 如以上任何一个答案为是，此受试者不能参加试验 | | |

**病历简况**

| **一般情况** | | | |
| --- | --- | --- | --- |
| 1.受试者登记号 |  | | |
| 2.入院日期 |  | | |
| 3. 年龄 |  | 4. 孕周 |  |
| 5. 孕产次 |  | 6. 胎数 |  |
| 7. 联系电话 |  | | |
| 既往史 | | | |
| □无 □有 | | | |
| 如有，请注明 |  | | |
| 家族史 | | | |
| □无 □有 | | | |
| 如有，请注明 |  | | |
| **一般情况检查** | | | |
| 1.体温（℃） |  | 2.脉搏（次/分） |  |
| 3.呼吸（次/分） |  | 4.血压（mmHg） |  |
| 5.身高（cm） |  | 6.体重（Kg） |  |
| **术中情况** | | | |
| 手术时间（min） |  | 失血量（ml） |  |
| 备注： | | | |

**用药记录**

**(antibiotics record)**

| 用药天数 | 用药次数 | 用药时间（24小时制） | 用药剂量 | 备注 |
| --- | --- | --- | --- | --- |
| 手术当天 | No.1 |  |  |  |
|  | No.2 |  |  |  |
|  | No.3 |  |  |  |
|  | No.4 |  |  |  |
| 术后1天 | No.1 |  |  |  |
|  | No.2 |  |  |  |
|  | No.3 |  |  |  |
|  | No.4 |  |  |  |
| 术后2天 | No.1 |  |  |  |
|  | No.2 |  |  |  |
|  | No.3 |  |  |  |
|  | No.4 |  |  |  |
| 术后3天 | No.1 |  |  |  |
|  | No.2 |  |  |  |
|  | No.3 |  |  |  |
|  | No.4 |  |  |  |
| 术后4天 | No.1 |  |  |  |
|  | No.2 |  |  |  |
|  | No.3 |  |  |  |
|  | No.4 |  |  |  |
| 术后5天 | No.1 |  |  |  |
|  | No.2 |  |  |  |
|  | No.3 |  |  |  |
|  | No.4 |  |  |  |
| 术后6天 | No.1 |  |  |  |
|  | No.2 |  |  |  |
|  | No.3 |  |  |  |
|  | No.4 |  |  |  |

**用药小结**

| 头孢硫脒总使用剂量 □□g 头孢硫脒使用时间 □□小时 |
| --- |
| 是否换药 □是 □否 若是，写明 |
| 换药使用时间 □□小时 |

**结果记录**

**(Outcome record)**

**1.体温记录**

**Temperature**

| 术后时间 | 体温 | 是否正常 | 若异常  是否有临床意义 |
| --- | --- | --- | --- |
| 6小时 |  | □是 □否 | □是 □否 |
| 12小时 |  | □是 □否 | □是 □否 |
| 24小时 |  | □是 □否 | □是 □否 |
| 48小时 |  | □是 □否 | □是 □否 |

**2.48小时后血常规检查记录**

**(BRT 48hours after CD)**

| 检查项目 |  | 是否正常 | 若异常  是否有临床意义 |
| --- | --- | --- | --- |
| WBC(10^12^ /L) |  | □是 □否 | □是 □否 |
| NEUT |  | □是 □否 | □是 □否 |
| Hb（g/L） |  | □是 □否 | □是 □否 |
| PLT(10^9^ /L) |  | □是 □否 | □是 □否 |

**3.子宫内膜炎诊断记录(**Endometritis)

| 诊断项目 | 是 | 否 |
| --- | --- | --- |
| 体温（术后24小时体温单次大于38.3℃或两次超过38℃） | □ | □ |
| 子宫和子宫旁组织压痛 | □ | □ |
| 宫颈口见大量脓性或血性混浊臭味分泌物 | □ | □ |
| **子宫内膜炎** | □ | □ |
| 备注 | | |

1. **切口感染诊断记录**Surgical site infection

| 诊断项目 | 是 | 否 |
| --- | --- | --- |
| 切口红肿 | □ | □ |
| 切口发热、压痛 | □ | □ |
| 切口化脓性分泌物 | □ | □ |
| **切口感染** | □ | □ |
| 备注 | | |

1. **尿路感染诊断记录**Urinary tract infection

| 诊断项目 | 是 | 否 |
| --- | --- | --- |
| 小便分析异常 | □ | □ |
| 侧腹疼痛 | □ | □ |
| 小便培养阳性 | □ | □ |
| **尿路感染** | □ | □ |
| 备注 | | |

1. **新生儿结果指标**Neonatal sepsis, Sepsis workup

| 诊断项目 | 是 | 否 |
| --- | --- | --- |
| 大便涂片菌群失调  若是，注明 □Ⅰ □Ⅱ □Ⅲ  球/杆比数值 □□□ | 此处  不填 | 此处  不填 |
| 新生儿败血症 |  |  |
| 送血标本培养 |  |  |
| 进NICU |  |  |

**不良反应/事件记录**

**(ADR record)**

| **不良反应/事件名称** |  |  |
| --- | --- | --- |
| **怀疑药物** |  |  |
| **日 期** | □□□□年□□月□□日 | □□□□年□□月□□日 |
| **不良反应/事件**  **过程描述** |  |  |
| **不良事件**  **严重程度** | - 轻 - 中 - 重 | - 轻 - 中 - 重 |
| **与药物**  **的关系** | - 肯定有关 - 很可能有关 - 可能有关 - 可能无关 - 无关 - 无关 | - 肯定有关 - 很可能有关 - 可能有关 - 可能无关 - 无关 |
| **采取的措施** | - 继续用药 - 减小剂量 - 暂停后又恢复 - 停用药物 - 对症治疗 | - 继续用药 - 减小剂量 - 暂停后又恢复 - 停用药物 - 对症治疗 |
| **转 归** | □ 消失  □ 缓解  □ 继续  □死亡 | □ 消失  □ 缓解  □ 继续  □死亡 |
| **纠正治疗** | □ 否 □ 是 | □ 否 □ 是 |
| **是否上报不良反应报告** | □ 否 □ 是 | □ 否 □ 是 |
| **因不良事件而退出试验** | □ 否 □ 是 | □ 否 □ 是 |

**出院记录**

**(Discharge record)**

| 出院日期 □□□□年 □□月 □□日 |
| --- |
| 出院诊断 |
| 住院天数 □□□天 |
| 住院费用 小写： ； 大写：□□□万□仟□佰□拾□元□角□分  药物费用 小写： ； 大写：□□□万□仟□佰□拾□元□角□分 |

**出院后随访记录**

**(Follow-up record)**

| 随访日期 □□□□年 □□月 □□日 | | | |
| --- | --- | --- | --- |
| 随访问题 | 是 | 否 | 备注 |
| 出院后至今体温正常 | □ | □ |  |
| 出院后至今有无感染症状 | □ | □ |  |
| 出院后至今服用其他抗感染药物 | □ | □ |  |
| 是否再次入院治疗 | □ | □ |  |

**出院后随访记录**

| 随访日期 □□□□年 □□月 □□日 | | | |
| --- | --- | --- | --- |
| 随访问题 | 是 | 否 | 备注 |
| 出院后至今体温正常 | □ | □ |  |
| 出院后至今有无感染症状 | □ | □ |  |
| 出院后至今服用其他抗感染药物 | □ | □ |  |
| 是否再次入院治疗 | □ | □ |  |

**研究完成情况总结**

| **受试者在试验期间是否有不良反应/事件发生？**　　　　　　　□是　　□否  **如果有不良反应/事件，是否均已解决？**　　　　　　　　　 □是　　□否  如否，应监测不良反应/事件直到稳定或解决。 |
| --- |
| **受试者是否按时完成了临床试验？**　　　　 　　 □是　　□否  如否，请填写以下项目： |
| **受试者终止试验日期：**□□□□年□□月□□日  **终止试验的主要原因是：**□   1. 不良事件(已填写不良事件表) 2. 失 访 3. 转 科 4. 转 院 5. 死 亡 6. 其它(请注明): |

**CRF表审核声明**

**(CRF audited statement)**

我已审阅过此病例报告表中的全部内容和数据，确认信息记录真实、准确，项目填写完整，符合试验方案的要求，特此声明。

试验中心负责人

□□□□年□□月□□日

**附页1**
