# Supplementary material for: Timing of Antibiotic Prophylaxis in Elective Caesarean Delivery: A Multi-Center Randomized Controlled Trial and Meta-Analysis
Source: PLoS One. 2015 Jul 6;10(7):e0129434. doi: 10.1371/journal.pone.0129434 (PMC4492889; doi:10.1371/journal.pone.0129434)
Supplement: S2 File — (DOC) [file pone.0129434.s002.doc]

Dear editor:

Thank your valuable suggestions.

We confirm that the clinical trial protocol we have included as Supplementary Information is the version that was submitted to and approved by our ethics committee before the trial began. We also uploaded the ethical review report and case report form which we have translated into English this time.

Secondly, we have completed the page numbers on the consort checklist.

Thank you for considering our paper to PLOS ONE.

Kind regards,

Lingli Zhang.

Department of Pharmacy, Evidence-Based Pharmacy Center, West China Second University Hospital, Sichuan University, Chengdu, China

E-mail: zhanglingli@sina.com

Tel: +8613547879789

**Timing of Antibiotic Prophylaxis in Elective Caesarean Delivery: a Multi-center Randomized Controlled Trial protocol**

**Background**

The current debate focused on the caesarean delivery was the choice of antibiotic and the timing of administration. China, the country with the highest rate of caesarean delivery in Asia, in contrast to other countries, recommended antibiotic prophylaxis post clamping umbilical cord in caesarean delivery instead of pre-incision. This study compared the effectiveness of timing of antibiotic prophylaxis, pre-incision and post-clamping umbilical cord in caesarean delivery.

**Design**

This multi-center randomized controlled trial with three parallel arms was conducted at three hospitals in western China (West China Second University Hospital, Nanchong central hospital and Sichuan women and children' s health hospital), from December January 1st, 2011, through to June 30th, 2012. Randomization was completed with SPSS 16.0-generated algorithm by an investigator with no clinical involvement in the trial. Participants were assigned randomly to pre-incision and after cord clamping in a 1 : 1 ratio. Treating assignments, kept in sealed opaque envelopes with only number labeled, were opened after the investigator evaluated the participant is eligible. Another investigator recorded patients in each group enrollment and patient assignment. Research assistant of trial and statisticians who were responsible for outcome recording and data analysis respectively were blinded to treatment assignment.

**Participants**

Subjects were eligible for the trial and were included in elective CD group if their gestational weeks were larger than 37 weeks. Exclusion criteria included cephalosporin allergy, exposure to any antibiotic agent two weeks before CD, the temperature above 37.5℃before CD, concomitant premature rupture of membrane, pernicious placenta praevia or the need for emergent cesarean delivery.

**Interventions**

Two grams of cefathiamidine (Guangzhou baiyunshan pharmaceutical Co. LTD) mixed with 100 mL of saline solution were used for antibiotic prophylaxis at most 0.5-2 hours before skin incision or after clamping umbilical cord according to the determined allocation. Another two grams of cefathiamidin were given 6 hours after CD. Participants were tracked through their hospital course and up to 6 weeks postpartum by telephone.

**Outcome**

**Primary outcome**

Endometritis was diagnosed if maternal temperature was above 38℃ on 2 separate occasions along with uterine fundal tenderness, tachycardia, or leukocytosis. Surgical site infection was diagnosed if there was purulent discharge, erythema, and induration of the incision site. Urinary tract infection was diagnosed by urine culture showing more than 100,000 colonies of a gram negative uropathogen. Puerperal morbidity was diagnosed if maternal temperature was above 38℃ on 2 separate occasions due to infection.

Neonatal sepsis was diagnosed by a positive blood culture. Organism, antibiotic resistances, and clinical course data were recorded. Sepsis workup and the place of admission of all neonates were determined by the neonatologists who were blinded to group assignment.

**Secondary outcome**

Body temperatures taken 6, 12, 24, and 48 hours after CD and results of blood routine test conducted 48 hours after CD were recorded by the trial assistant (CMH). Abnormal temperature was defined as above 37.5℃. Neonatal stool bacterial flora was also examined and recorded by the trial assistant (CMH). The status of neonatal gut flora was examined by direct rapid smear method and divided into four categories as normal, mild abnormal, moderate abnormal, and serious abnormal. Normal was defined as the situation that Gram-positive and negative bacilli and cocci were within the normal range. Mild abnormal was defined as the reduction of Gram-positive bacteria and proliferation of Gram-negative bacilli or positive cocci. Moderate abnormal was defined as the significant reduction of Gram-positive bacteria, proliferation of Gram-negative bacilli or positive cocci, and inverse ratio of bacilli and cocci. Serious abnormal was defined as the situation that only one dominant bacteria or fungi was observed and other flora was suppressed.

**Statistical analysis**

Power was calculated using incidence of maternal infectious morbidity which was reported to be 17% in the literatures. Using a power of 0.80 and an alpha of 0.05, 190 subjects per arm were calculated to be necessary to detect a 50% decrease in overall infectious morbidity of participants given preoperative antibiotic prophylaxis. In consideration of 10% loss of follow-up rate, a total of 410 patients were required, with 205 in each group.

Intention-to-treat and per-protocol analysis were used to analyze the data of trial. Demographic and other characteristics at trial entry will be tabulated using the intention-to-treat analysis. The other outcomes were analyzed by per-protocol analysis. Meta-analysis and trial sequential analysis will be conducted for all primary outcomes. Statistical software SPSS 16.0, reviman 5.1 and Trial Sequential Analysis (version 0.9) were used for analyses. Continuous parameter was analyzed by student’s t-test. Discrete data was analyzed with chi-square test. Results with a p value<0.05 were considered statistically significant.

**Data management**

Quantitative data will be collected from both the intervention and control arms of the study by study researchers in the hospital. Patients were followed up after discharge by telephone. Researchers will be trained together to ensure that similar methods are used in all centers.

**Study monitoring**

An independent Data Monitoring Committee (DMC) has been established, whose responsibility is to train investigators, collaborators and administrative staff and review the trial’s progress. Meetings of the committee will be arranged periodically, as considered appropriate by the Chair. Collaborators and all others associated with the study may write to the DMC via the Trial Coordinating Center, to draw attention to any concern they may have about the possibility of harm arising from the treatment under study.
